# Protein Content in the Diet Influences Growth and Diarrhea in Weaning Piglets

**DOI:** 10.3390/ani13050795

**Published:** 2023-02-22

**Authors:** Rosa Marchetti, Valerio Faeti, Maurizio Gallo, Massimo Pindo, Davide Bochicchio, Luca Buttazzoni, Giacinto Della Casa

**Affiliations:** 1 Council for Agricultural Research and Economics, Research Centre for Animal Production and Aquaculture, Via Beccastecca 345, 41018 Modena, Italy; 2 Associazione Nazionale Allevatori Suini, Via Nizza 53, 00198 Rome, Italy; 3 Fondazione Edmund Mach (FEM), Research and Innovation Centre, Via E. Mach 1, 38010 Trento, Italy; 4 Council for Agricultural Research and Economics, Research Centre for Animal Production and Aquaculture, Via Salaria 31, 00015 Rome, Italy

**Keywords:** piglet, post-weaning diarrhea, dietary protein, fecal microbiota, feces composition

## Abstract

**Simple Summary:**

Weaning (that is, removal from the sow) and the following two months are the riskiest periods in a pig’s life, especially for pig’s gastrointestinal health. The change in diet due to the suspension of the mother’s milk, accompanied by an acceleration of both morphological and enzymatic maturation of the intestinal mucosa of the piglets, can worsen digestion and absorption. In this context, the protein requirement of piglets, which are in a phase of rapid growth, may be greater than the intestine’s ability to digest proteins. Undigested proteins are the best pabulum for the proliferation of the pathogenic bacterial flora that causes diarrhea. Since these problems can no longer be resolved with prophylactic use of antibiotics, the best balance between intestinal health and growth performance must be found. A diet low in crude protein and supplemented with synthetic amino acids can help achieve this goal.

**Abstract:**

The aim of this research has been to assess the effect of the dietary protein level on piglet growth and post-weaning diarrhea (PWD) incidence. Piglet fecal microbiota and feces composition were also assessed. The experiment was carried out on 144 weaned piglets (Duroc × Large White; 72 piglets per treatment) and lasted from weaning (at 25 days of age) until the end of the post-weaning phase (at 95 days). Two dietary protein levels were compared: high (HP; 17.5% crude protein on average, during the experiment) and low (LP; 15.5% on average). Lower (*p* < 0.01) average daily gain and feed conversion ratio were observed in LP piglets in the first growth phase. However, at the end of the post-weaning period, the growth parameters were not significantly different in the two diets. Diarrhea scores were lower in piglets fed LP diets than in piglets fed HP diets (28.6% of the total vs. 71.4% in the HP piglets). *Fibrobacteres*, *Proteobacteria,* and *Spirochaetes* were more abundant in the feces of the piglets fed LP diets. Feces nitrogen content was lower in piglets fed LP diets. In conclusion, low protein levels in the diet can reduce the incidence of PWD while only marginally affecting growth parameters.

## 1. Introduction

The period between weaning (i.e., removal from the mother) and reaching the bodyweight for transfer to the fattening boxes (at about three months of age and 35–40 kg of body weight) is more delicate from the point of view of health and functioning of the piglet digestive system. Among the numerous factors that can intervene to destabilize the delicate balance of the piglet’s intestine, linked to an acceleration phase of both morphological and enzymatic maturation, diet-linked factors undoubtedly play a fundamental role [1].

Among the dietary factors, the quantity of protein fed to piglets plays a leading role. In fact, in this phase, the coverage of nutritional needs requires a protein level higher than the digestive potential of the piglet. According to Kim et al. [2], a protein level of between 21.5% and 24% is required for modern fast-growing lines, a level that, in fact, is higher than the piglet’s digestive capacity; these authors suggest a protein level not higher than 18% in the first days after weaning and with a consistent addition of synthetic amino acids. De Lange et al. [3] pointed out that low protein levels are beneficial for the gut health of piglets because the presence of undigested proteins, as it can occur with high dietary protein levels, allows the proliferation of a bacterial flora producing toxins capable of altering the intestinal barrier. This alteration implies: the colonization of the intestinal epithelium by pathogenic microorganisms; the acceleration of the production of enterocytes that, being immature, have an exudative rather than absorbent attitude; greater ease of crossing the cellular barrier by specific bacterial toxins (edema disease). Zhang et al. [4] highlighted that high-protein diets increase the microbial fermentation of proteins, peptides, and amino acids. According to Gao et al. [1], high protein levels favor the production of ammonia and branched-chain-fatty acids and, therefore, the proliferation of pathogenic bacterial flora, while low protein levels favor the production of short-chain fatty acids (SCFAs), primarily butyric acid, which favors the proliferation of beneficial bacterial flora. The same authors, comparing two protein levels (17% vs. 30%), both obtained exclusively with casein, highlighted that with a high protein level, the bacterial diversity of the microbiota is reduced. The increase in ammonia can negatively affect the formation of intestinal epithelial cells [5]. The reduction of beneficial *Lactobacilli* that accompanies the maturation of the pig’s intestine and the variations in the buffering effect of pH due to protein fermentation can make the intestinal environment more susceptible to the emergence of opportunistic pathogens, such as *Bacteroides* and *Clostridium* species [6]. Opapeju et al. [7] compared four diets administered to piglets with an initial weight of about 6.5 kg: control feed with 21% crude protein (CP); feed with 19% CP and deficient in isoleucine; 19% raw protein feed supplemented with synthetic isoleucine to reach the isoleucine level of the control feed; 17% raw protein feed supplemented with isoleucine and valine to reach the ratio indicated by the ideal protein. The control group showed better production performance in terms of growth and conversion index, although they showed softer feces, a greater amount of ammonia in the feces, and a greater depth of the crypts of the intestinal mucosa, indicating an acceleration of production of enterocytes; essentially, better production performance, but greater susceptibility to the onset of a syndrome affecting the gastrointestinal system.

This situation of precarious equilibrium could be easily kept under control only with targeted antibiotic prophylaxis, which, however, is no longer allowed, and even the spaces for metaphylaxis, albeit careful, become increasingly restricted. Therefore, it is necessary to identify feeding strategies that reduce the risk of the appearance of alterations in the gastrointestinal function of the pig [8] and reserve the use of antibiotics for clinically overt pathological situations.

Knowledge of the relationship between pig microbiota and diet can be used to orient the intestinal microbial dynamics in the desired direction by diet manipulation [9]. The microbiota of healthy piglets susceptible to post-weaning diarrhea (PWD) has been the subject of numerous investigations [10,11], which highlighted how the state of health of the pig and its susceptibility to diseases, such as PWD, can be related to the change in the composition of the microbiota during the early stages of growth.

All factors affecting PWD susceptibility also affect microbiota composition. Among these, pig feeding plays a primary role. Heo et al. [12] observed a reduction in PWD in piglets challenged with an enterotoxigenic strain of *Escherichia coli*, when fed with lower protein levels. Rist et al. [13] suggested reducing proteins and increasing fermentable carbohydrates in the diet to reduce harmful protein fermentations. Luise et al. [14] showed that lower dietary protein levels could reduce the intestinal fermentation of undigested proteins and the consequent risk of diarrhea. The aim of this research has been to assess the effect of the dietary protein level on piglet growth and PWD incidence. Piglet fecal microbiota and feces composition were also assessed to support the understanding of the results.

## 2. Materials and Methods

### 2.1. Animal Ethics

All animal procedures were performed in strict accordance with the Code of Ethics of the World Medical Association (https://ec.europa.eu/environment/chemicals/lab_animals/legislation_en.htm. Accessed on 19 February 2023).

### 2.2. Experimental Design

The experiment was carried out at our experimental pig farm in San Cesario sul Panaro (Modena, Italy), on Duroc Italiana × Large White Italiana crossing lines. A total of 144 weaned piglets was used, half barrows and half females. The experiment lasted from weaning, when the age of the piglets was 25 ± 1.5 days, until the end of the post-weaning phase, corresponding to 70 days of trial and 95 days of age of the animals (Table 1).

Two dietary protein levels were compared (Table 2): high (HP) and low (LP), in two growth phases characterized by different diet compositions. The diet composition (Table 3) was changed 25 days after the start of the experiment, at the expected piglet body weight of 15 kg (and actual weight of 17 kg). Synthetic amino acids were also supplemented to ensure a balanced feed formulation. Specifically, total lysine levels in the first and second feeding periods were set at 1.40% and 1.20% of the feed, respectively. The percentages of methionine, cystine, threonine, and tryptophan were balanced according to the proportion of the ideal protein [15] with the addition of synthetic amino acids.

After separation from the mother, the piglets were housed in cages of 12 individuals each, distributed as evenly as possible within each cage by body weight, age, and litter of origin. Since it was not possible to accommodate 12 cages (3.3 m^2^ each) within the same room, the males were housed in one room (6 cages) and the females in another (6 cages). In this way, it was necessary to accept that we were dealing with a confused effect (room and sex), while the factor of interest of the experiment (protein level) was homogeneously represented in both rooms. Each cage was equipped with a hard-plastic floor. The complete feed was administered ad libitum in a hopper feeder with 4 places to ensure sufficient access to feed for all piglets; in each cage, there was a nipple drinker. The temperature of the air in the rooms was 23 °C ± 1 °C, whereas the humidity was not controlled.

Forty-six days after weaning, the piglets were moved to larger pens in the fattening area and housed in 12 pens (9 m^2^ each) of 12 piglets each. The floor was thermally insulated concrete. The pens were arranged in 2 rows of 6 pens (3 adjacent pens for males and 3 adjacent pens for females), one row for each protein level. The animals were given 1 kg of feed per animal and per day in two daily meals. In this phase, the administration of feed was limited to reduce the risk of diarrhea related to the stress of the change of housing. In the first two days, the meal was dry and distributed on the ground, gradually passing to a wet meal in the trough over the next three days. In each pen, the water was still available through a nipple drinker. The assignment of the treatment to the pens followed the criterion of minimizing the possibility of mixing feces from pigs fed different protein levels. In the fattening area, 21 °C was always ensured during the experiment.

The effects of dietary protein level on piglet growth, health status, and feces microbiota were considered for the following periods (Table 1):

- Period I: From the start of the experiment until the change of feed;
- Period II: From the change of feed until the change of housing;
- Period III: From the change of housing to the end of the post-weaning period (end of the experiment).

### 2.3. Growth Parameters

The animals were weighed individually at the start of the experiment, at the day of diet change, at the day of change in housing, and at the end of the experiment. For each period, average daily gain (ADG = [body weight at the end of the period—body weight at the beginning of the period]/day), average daily feed intake (ADFI = feed consumption in the period/day), and feed conversion rate (FCR = ADFI/ADG) were calculated. Since feed consumption was known only at the cage/pen level, the values of the ADFI and FCR variables were not known at the individual level and thus were calculated at the cage/pen level. For homogeneity, the ADG values, although individually known, were also processed at the cage/pen level, with six replicates in total for each treatment.

### 2.4. Diarrhea Scores and Corrective Interventions

The health of the piglets was monitored daily. A score was assigned to each cage/pen based on the number of cases and the extent (mild, medium, severe) of diarrheal phenomena in piglets, visually assessed from the consistency of the feces (Table 4). For each growth period, individual diarrhea scores were summed for each treatment and related to the period’s total diarrhea score.

Piglets suffering from diarrhea before the change of diet (Table S1) were treated (Table S2) parenterally with the antibiotics enrofloxacin or marbofloxacin. No antibiotics were administered between the time of the diet change and the date of transfer to the pens because there were no cases of diarrhea. After the transfer, all individuals were treated orally with colistin sulfate, starting 5 days after the transfer and for 8 days. It was decided to resort to oral mass therapy because the appearance of overt diarrhea (score 4 or higher) occurred five days after the moving, in four out of six boxes of the HP treatment and in one out of six boxes of the LP treatment (Table S3). No treatment was applied in the last 12 days before the third and final sampling of feces.

### 2.5. Feces Chemical Characterization

Feces samples were collected at the end of each growth period by piglet rectal ampoule stimulation. For each cage/pen, individual samples were pooled into a composite sample. Samples were immediately frozen and stored at −20 °C until analysis.

Dry matter, organic matter, total (Kjeldahl) nitrogen and ammonium nitrogen contents, and pH, were determined according to the APHA methods [16]. Crude fiber (CF) and fiber fractions were determined on fecal samples dried at 60 °C. Crude fiber was determined according to [17]. Hemicellulose and cellulose concentrations were estimated by determining neutral-detergent (NDF) and acid-detergent (ADF) fiber fractions and acid-detergent lignin (ADL) according to [18]. The difference between NDF and ADF is an estimate of the hemicellulose content; that between ADF and ADL of the cellulose content.

For volatile fatty acid determination, 1 g of the sample was diluted with 3 mL distilled water, then centrifuged at 4000 rpm for 15 min. The supernatant was used for the analyses. Half mL of sample supernatant was added to 0.25 mL 4% H_3_PO_4_ and 0.25 mL internal standard to a final volume of 1 mL. A microliter of this mixture was injected in the injection port of the gas-chromatograph (Shimadzu GC 2010 Pro), equipped with a NukolTM capillary column (Supelco, cat. no. 24107), 30 m × 0.25 mm internal diameter, 0.25 μm film thickness. Total volatile fatty acid content (mg L^−1^) was calculated as the sum of the individual concentrations of acetic, propionic, butyric and iso-butyric, valeric and iso-valeric, and caproic and iso-caproic acids.

### 2.6. DNA Extraction, Library Construction, and Sequencing

Total DNA was extracted from the fecal samples after thawing using the QIAamp PowerFecal Pro DNA Kit (QIAGEN, The Netherlands) according to the manufacturer’s instructions. The V3-V4 regions of the 16S rRNA gene were amplified using the 341F (5′-CCTACGGGNGGCWGCAG-3′) and 805Rmod (5′-GACTACNVGGGTWTCTAATCC-3′) (based on [19], with degenerate bases) primers.

Library construction and sequencing were performed at the Sequencing Platform, Fondazione Edmund Mach, Italy. More in detail: each sample was amplified by PCR using a 25 µL reaction mixture with 1 µM of each primer. More in detail, 12.5 µL of 2× KAPA HiFi HotStart ReadyMix and 10 µL forward and reverse primers, were used in combination with 2.5 µL of template DNA (5 ng/µL). The PCR reactions were carried out by GeneAmp PCR System 9700 (Thermo Fisher Scientific) and the following cycling conditions: initial denaturation step at 95 °C for 3 min (one cycle); 25 cycles at 95 °C for 30 s, 55 °C for 30 s, 72 °C for 30 s; final extension step at 72 °C for 5 min (1 cycle).

The amplification products were checked on 1.5% agarose gel and purified using the CleanNGS beads (CleanNA, Waddinxveen, The Netherlands) following the manufacturer’s instructions. Afterward, a second PCR was used to apply dual indices and Illumina sequencing adapters Nextera XT Index Primer (Illumina), by 7 cycles PCR (16S Metagenomic Sequencing Library Preparation, Illumina). The amplicon libraries were purified using the CleanNGS beads (CleanNA, The Netherlands), and the quality control was performed on a Typestation 2200 platform (Agilent Technologies, Santa Clara, CA, USA). Finally, all barcoded libraries were pooled in an equimolar way and sequenced on an Illumina^®^ MiSeq (PE300) platform (MiSeq Control Software 2.5.0.5 and Real-Time Analysis software 1.18.54.0). A total of 3.093.548 raw reads were detected across the samples by the Illumina MiSeq sequencing platform (PE300) (Illumina, Santa Monica, CA, USA).

### 2.7. Statistical Analysis

The estimate of the effect of the protein level in the diet on growth performance was carried out by means of a one-factor analysis of variance. The effect of the sex/room factor was included in the block effect. Values of F with *p* > 0.05 were considered not significant (NS).

The analysis of variance (ANOVA) for the effect of diet on diarrhea incidence was applied to the sum of the scores of the period for each treatment replication. Since in the second period the sum of the scores was very low, for the purposes of ANOVA, the first two periods were merged into a single period.

Two-way ANOVA (fixed sources of variation: time of sampling, protein level, time of sampling × protein level) was applied to operational taxonomic units (OTU) percentages to estimate the effect of diet and growth period on the relative abundance of phyla, families, and genera in the analyzed samples, using the procedure MIXED, SAS language [20]. A threshold equal to 0.1% of the total reads was adopted to include the phylum, family, or genus in the analysis. Mean multiple comparisons were performed using the statement LSMEANS and the Tukey HSD test.

The same two-way ANOVA design was applied to the statistical analysis of feces composition.

### 2.8. Bioinformatic Analyses

Data were pre-processed using the MICCA v. 1.7 [21] pipeline and rarefied to an equal depth of 45.225 reads per sample. OTUs were created de novo by clustering sequences with 97% sequence identity and classified using the RDP [22] software version 2.11.

The alpha diversity of the populations, the relative abundance (%) of microbial components down to the family and sex level, and their grouping based on the sources of variation were estimated. Alpha diversity is an index of the richness (number) and diversity (relative abundance) of OTUs in a population. The richness of species is indicated by the total number of OTUs (“Observed”) in the microbial community: the higher the number, the more species are present. The CHAO1 index estimates the richness of species, giving more weight to the less abundant ones. The value of CHAO1 is at least equal to “Observed” and increases as the number of rarer species increases. It can be calculated as follows:

CHAO1 = S_obs_ + F_1_(F_1_ − 1)/(2 × (F_2_ + 1)) (1)

where S_obs_ is the number of observed species and F_1_ and F_2_ are the count of singletons and doubletons, respectively.

The Shannon index is calculated as follows:

Shannon Index = −∑(p_i_ ln(p_i_)) (2)

where: Σ is the summation from 1 to the total number of OTUs, and p_i_ is the proportion of the community represented by the OTU i. It increases with increasing species richness, uniformity, and uncertainty of the estimate.

The samples were grouped for compositional similarity (beta diversity) using Principal Coordinate Analysis (PcoA), which is a multivariate method of data analysis used to explore and to visualize similarities or dissimilarities of data [23].

## 3. Results

### 3.1. Growth Performance

The piglets fed HP level showed a greater ADG (*p* < 0.01) than those fed LP (Table 5) in the first 25 days of the experiment until the change of diet, while the increases were the same for the two protein levels in the period from the change of diet to the change of housing. The difference remained significant (*p* < 0.05) during the entire phase in the cages. The FCR value was also significantly better in HP. Conversely, ADFI was not significantly different in piglets fed different protein levels. In the pen housing, no differences were detected between the two treatments as the feed was rationed. The body weight of the piglets at the end of the post-weaning period was not different in the two diets.

### 3.2. Health Status

Diarrhea cases started to occur in the early post-weaning period (Table S1). They were concentrated in the period between weaning and change of feed (Period I; score summation: 107 out of 234, i.e., 45.7% of the total; Table 6) and in the period between change of housing and the conclusion of the post-weaning period (Period III; 121 out of 234: 51.7% of the total). In Period II, overall, feces had normal consistency, as well as in the first and last part of Period III (Tables S1 and S3).

The overall incidence of diarrhea in the LP treatment was 28.6% of the total (67 out of 234 scores) and 71.4% in the HP treatment. Specifically, from weaning until feed change (Period I), the diarrhea scores in the LP treatment were 15.0% of the total (16 out of 107); after feed change (Period II), they were 16.7% of the total (1 out of 6), while after the change of housing (Period III) they rose to 41.3% (50 out of 121). The effect of the dietary protein level on diarrhea score summations was highly significant (*p* < 0.01) until change of housing (Period I + Period II), whereas it was not significant (*p* = 0.140) from the change of housing until the end of the experiment (Period III).

Poor feces consistency was noted in all the boxes in the HP treatment, starting from the twelfth day of the experiment (Table S1). The phenomenon initially extended to the whole pen in three out of six pens and then progressively became reduced, presumably due to therapeutic interventions (Table S2), until it disappeared two days after the change of feed.

### 3.3. Composition of the Fecal Microbiota

**Figure 1:**
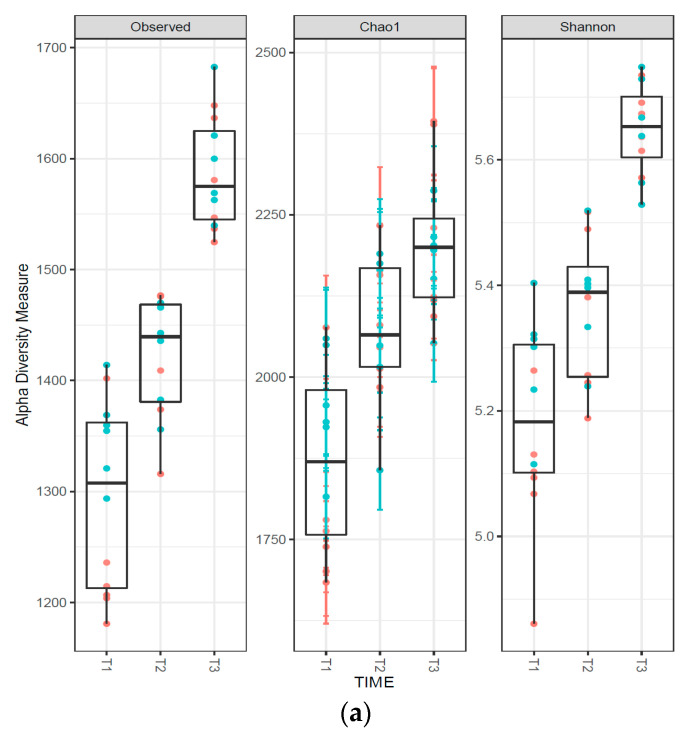

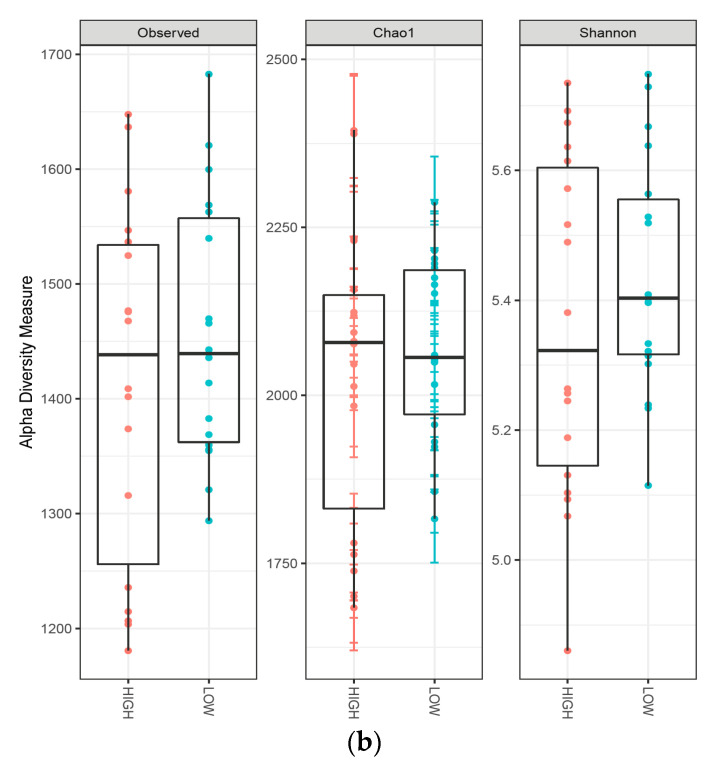
Box plots of the alpha diversity indexes (observed, Chao1 and Shannon) as a function of (**a**) sampling time and (**b**) protein level (high, red dots; or low, blue dots). T1, T2, and T3 correspond to fecal samplings after weaning and before the change of feeding, before the change of housing, and at the end of the experiment (end of the post-weaning period), respectively.

The alfa diversity of the microbiota (intra-sample diversity) increased after the change of feed and further increased after the change of housing (Figure 1a), while it was not influenced by the protein level in the diet (Figure 1b).

**Figure 2:**
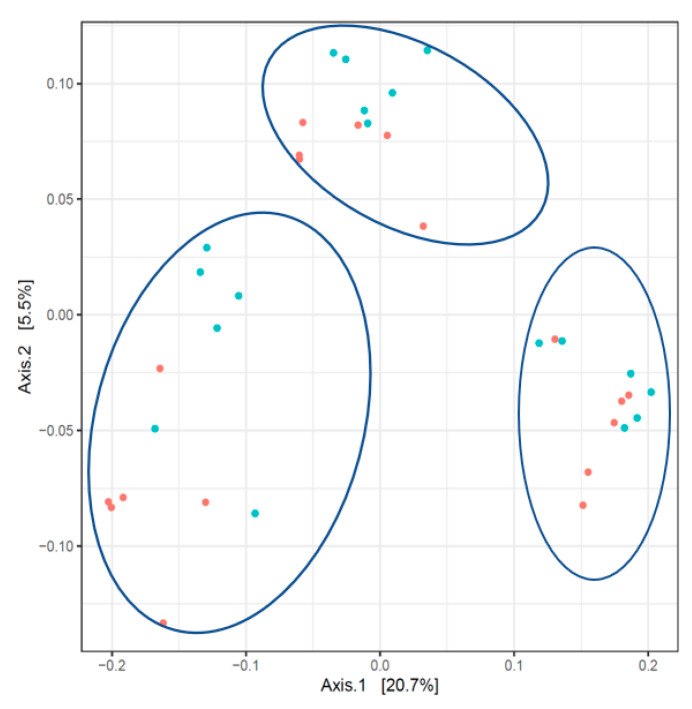
Principal coordinate analysis of the piglet fecal microbiota. Red dots: high protein level, blue dots: low protein level. Contour lines highlight the 3 sampling times, T1, T2, and T3, from left to right.

The analysis of beta diversity (inter-sample diversity) using the PCoA tool allowed the clustering of the fecal samples into three groups, corresponding to the three times of sampling (Figure 2). The protein level in the diet allowed only partial separation of the treatments.

The most represented phyla in the piglet fecal samples (Table 7) were *Bacteroidetes* and *Firmicutes*, which together accounted for 90.6% of reads. The relative abundance of phyla changed in relation to the sampling date: at the first sampling, 25 days after weaning, *Firmicutes* and *Actinobacteria* were more abundant than in the two subsequent samplings, whereas the other classified phyla: *Bacteroidetes*, *Spirochaetes*, *Proteobacteria*, and *Fibrobacteres* were more abundant in the two samplings following the first, without significant differences between the second sampling, at the end of a period characterized by a change of feed, and the third sampling, at the end of a period that had begun with the change of housing. *Fibrobacteres*, *Proteobacteria,* and *Spirocheaetes* were, on average, more represented in the feces of piglets on a low-protein diet, whereas *Firmicutes* were more abundant in the feces of piglets on a high-protein diet.

Overall, the most represented bacterial families were: *Prevotellaceae*, *Lachnospiraceae*, *Ruminococcaceae,* and *Porphyromonadaceae* (65.1% of the total reads). While the influence of the sampling time was evident, the same cannot be said for that of the treatment (Table S4). Low protein levels in the diet were associated with a higher abundance of *Fibrobacteraceae*, *Succinivibrionaceae*, *Sutterellaceae*, and *Spirochetaceae*, whereas *Firmicutes*-belonging families prevailed in the HP fecal samples: *Lachnospiraceae* and *Eubacteriaceae* were more abundant along all the post-weaning period, whereas *Erisipelotrichaceae, Clostridiaceae 1,* and *Peptostreptococcaceae* were more abundant only in the first sampling event. *Lactobacillaceae* were the only *Firmicutes* more abundant in the feces of piglets on a low-protein diet and only in the first sampling event (significant interaction effect: Sampling time × Protein level). A few classified families were little or not at all influenced by both the period of growth and the protein level; among these, *Enterobacteriaceae*.

Among the classified genera, the most abundant were *Prevotella* (27.2%), followed by *Clostridium sensu strictu* (3.9%), *Lactobacillus* (3.3%), *Alloprevotella* (3.2%), *Treponema* (3.1%) (Table S5). Selected genera were more represented in the families significantly influenced by the dietary protein level, alone or in interaction with the sampling time (Table 8). *Lactobacillus* and *Treponema* were more represented in the LP fecal microbiota, whereas *Roseburia*, *Blautia,* and some *Clostridium* genera were more abundant in HP.

### 3.4. Feces Composition

Almost all the traits analyzed varied significantly over time (Table 9). More specifically, the concentration of dry matter, organic matter, crude fiber, and fiber fractions increased over time, whereas total and ammonium nitrogen concentrations decreased. The pH slightly increased after the feed change. The dietary protein level also affected the feces composition. Piglets fed low protein had feces that were richer in dry and organic matter and lower in total and ammonium nitrogen all over the experiment period. Even though crude fiber concentration was not affected by the dietary protein level, however, the fiber fractions were. In fact, hemicellulose and cellulose concentrations were higher in the LP fecal samples.

As for volatile fatty acids, the concentrations of propionic, valeric, and especially isovaleric acid slightly increased after the change of feeding. No effect on the protein level was detected, except for the isovaleric acid concentration, which tended to be lower in low-protein diets.

## 4. Discussion

### 4.1. Dietary Protein Level, Growth Performance, and Susceptibility to PWD

In our experiment, the low protein level negatively affected the growth of the piglets (ADG and FCR) only in the first post-weaning period (up to the change of diet), whereas it had a clear positive impact on diarrhea intensity reduction. Literature on the matter reports contrasting results, as also highlighted by Wang et al. [24]. Heo et al. [12] reported a higher incidence of PWD when feeding weaned piglets at a high (24.3%) compared with a low (17.3%) CP diet. Wen et al. [25] observed a higher incidence of PWD for piglets fed higher protein levels (up to 23%). Rattigan et al. [26] obtained contrasting results by working in sanitary vs. unsanitary conditions. Reducing the dietary CP level did not affect growth performance; however, in sanitary conditions, it increased the *Enterobacteriaceae* abundance in the colon and the incidence of diarrhea occurrence, whereas the opposite occurred in unsanitary conditions. Limbach [27] tested the effect on growth and PWD incidence in piglets fed soybean-maize diets at three protein levels (22% AA-balanced, 19% AA-balanced, and 16% protein AA-unbalanced) and concluded that low CP diets may be used for the initial post-weaning period to reduce piglet susceptibility to PWD without largely impacting growth performance. Lynegaard et al. [28] overall reduced CP in the first weaning phase (6–9 kg) from 19.1% to 16.6 and 14%, and from 18.4% to 16.2–17.4%, in the second phase (9–15 kg), depending on the treatment, while they left the protein almost constant in the third phase (15–30 kg) of growth. Under these conditions, they observed a decrease in PWD incidence for treatments with reduced dietary protein, as well as lower ADG and FCR values in piglets fed lower protein levels up to 15 kg body weight. The disadvantage, especially in terms of ADG, also remained in the following period. In their experiment, however, the reduction of CP in the early post-weaning growth period was more pronounced than in our experiment. According to [29], CP levels too low (i.e., 3% below average: 17%) are considered harmful in consideration of maladaptive changes to small intestinal morphology and pepsin activity in weaned piglets.

### 4.2. Piglet Fecal Microbiota in the Post-Weaning Period

The observed increase in the microbiota’s alfa diversity during piglet growth has also been reported by other authors [30], and it can be linked to changes in diet as well as to the progressive maturation of the intestinal system [31]. On the contrary, dietary protein levels have been reported not to influence alpha diversity in pigs [32,33]. Our results confirm these findings.

The most represented phyla (Table 7) are *Bacteroidetes*, *Firmicutes*, *Spirochaetes,* and *Proteobacteria* (96.9% relative abundance), and the most abundant bacterial families: *Prevotellaceae*, *Lachnospiraceae*, *Ruminococcaceae,* and *Porphyromonadaceae* in the piglet fecal samples of this experiment are those recurring in the feces of healthy piglets [34,35]. *Clostridium sensu stricto*, *Roseburia*, *Paraprevotella*, *Clostridium XIVa*, and *Blautia* have been reported as major representative genera after weaning [36,37].

In general, the results in the literature refer to piglets stressed on purpose or younger than those in this experiment. The considered microbiota is more often that of the intestinal system, different from the fecal one [38]. In our experiment, we considered the microbiota of the feces of piglets raised in protected conditions, which should be the most likely or, in any case, desired in real farms. This protection has been applied both at the environmental level (attention to the absence of causes of stress) and at the health level (preventive interventions with drugs). This may explain the low presence of *Enterobacteriaceae*, which is normally associated with stressful conditions.

### 4.3. Dietary Protein Level, Composition of the Fecal Microbiota, and PWD Susceptibility

The effect of dietary protein level on the composition of gut microbiota has mainly been studied for finishing pigs. Moderate protein levels in the diet have been found to modify the gut microbiota composition and to improve the ileal barrier function [39,40]. On the contrary, information on piglets is scarce, especially when referring to the composition of the fecal microbiota. In our experiment, differences in the composition of the fecal microbiota due to the protein level in the diet were found above all in the first sampling, that is, at the end of the first period after weaning and before the change of feed, just when the effect of protein intake on the incidence of diarrhea phenomena was most evident. Given this coincidence, we can think of associating the higher susceptibility of the piglets to PWD with the greater presence of *Firmicutes*-belonging families (with the exclusion of *Lactobacillaceae*), which were more abundant in T1 in the fecal microbiota of piglets fed HP diets. Conversely, a lower incidence of PWD can be associated with the prevailing abundance in LP diets of families belonging to *Lactobacillaceae* (gen. *Lactobacillus*), *Fibrobacteraceae* (gen. *Fibrobacter*), *Succinivibrionaceae*, and *Spirochetaceae* (gen. *Treponema*).

Yang et al. [30] compared the fecal microbiota of healthy piglets and that of diarrheal piglets in three stages of growth: lactation, intermediate stage, and weaning (solid diet for piglets) and noted that, with the transition to solid feeding, the incidence of *Lactobacillus* and *E*. *coli* decreased while that of *Prevotella* increased. They related reduced numbers of *Bacteroides*, *Ruminococcus*, *Bulleidia*, and *Treponema*, which are responsible for the digestion of solid foods, to the onset of post-weaning piglet diarrhea.

The genera prevalent in the fecal microbiota of piglets fed HP diets, such as *Roseburia*, *Blautia*, *Eubacterium*, and selected *Clostridium* species, are commonly found in piglet fecal microbiota and are all fermentative. Their greater abundance in the HP feces can be the consequence of the incomplete digestion of complex fermentable substrates because less digestible feed components are more available for microbial activities in the gut terminal tract. The greater presence of *Clostridia* in the microbiota of piglets fed HP diets can be related to their ability to metabolize amino acids [4]. Conversely, the increased presence of *Fibrobacter* in low-protein diets can be related to the availability of higher amounts of undigested cellulose in the final intestinal tract. In fact, *Fibrobacter* is a fibrolytic bacterial species commonly present in the pig intestinal microbiota [41], although it is a typical colonizer of the rumen. *Fibrobacter* uses glucose, cellulose, and cellobiose as carbon and energy sources with the production of succinate, acetate, and formate [42]. *Fibrobacter* and *Treponema* have been reported as more abundant in diets of ruminants fed with higher amounts of lignocellulosic components [43].

### 4.4. Dietary Protein Level and Feces Composition

Feces composition clearly depends on the type of food ingested and on the use that the animal can make of the food. The less digestible the food, and the worse the digestive abilities of the animal, the higher the quantity of feed that remains undigested in the feces. Higher crude fiber contents accompanying the change of feed can be the reason for worse digestibility: a higher crude fiber content was, in fact, found in the piglet feces at the second and third sampling (i.e., at the change of housing and at the end of the post-weaning period).

An important side effect of the reduction in dietary protein was the lower concentration of nitrogen in the feces, possibly because a greater quantity of ingested nitrogen was assimilated instead of wasted. This result has been reported by several authors. Zhao et al. [44] found a significant reduction in N excretion in 90-day-old pigs fed protein diets 3.5% lower than standard. Yang et al. [45] observed a linear decrease in fecal total nitrogen for decreasing dietary protein levels in the diet of growing pigs.

Other components in the diet may interfere with nitrogen metabolism. In fact, in maize, the nitrogen-free extractives consist of starch, whereas in soybeans, the starch is less than 1%, and soluble carbohydrates are mainly sucrose, raffinose, and stachyose [46]. High amounts of raffinose and stachyose from soybean in the diet are supposed to reduce the digestibility of nitrogen and amino acids in growing pigs [47]. Therefore, in HP-fed piglets, the greater nitrogen excretion can also derive from a lower digestibility due to the higher percentages of soybean oligosaccharides. Interestingly, Zhang et al. [48] showed a higher incidence of diarrhea cases in piglets fed with soybean flour as is or with added stachyose compared to the control consisting of corn with concentrated soybean protein.

Short-chain fatty acids are final metabolites of the intestinal microbiota, produced mainly in the large intestine, where they are used by mono-gastrics as a source of energy. The energy contribution of the SCFA in pig metabolism is important. Weaning has been reported to affect the concentrations of SCFA in the intestine [49]. In our experiment, the dietary protein level did not affect the SCFA content, apart from the case of isovaleric acid, which was less abundant in LP diets.

Some confirmations and some interesting insights emerge from this experience. The first of these is whether the body weight differences found in the experiment cancel out during the subsequent stages of growth of the animals and, once this has been established, how far one can go with the reduction of the protein content in particularly critical phases (first 15 days after weaning, sudden changes in the environment) without compromising the subsequent productive career. The second interesting point is to investigate the evolution of the microbiota to identify moments in which an analysis of this can serve as an indicator of the evolution of the state of health of the gastrointestinal system. From our results, it appears that the phase immediately following weaning is the most subject to changes in the fecal microbiota composition and most suitable for earlier identification of possible stress conditions. Everything must be understood in terms of optimizing individual and mass therapeutic interventions in order both to improve the profitability of breeding and to reduce the risk of the appearance of antibiotic-resistance phenomena.

## 5. Conclusions

This research confirms that a reduction in the protein content of feed can reduce the appearance and severity of gastrointestinal syndromes in piglets in particularly stressful stages of rearing (removal from the mother, change of housing) while only marginally affecting growth performance. Low-protein diets, resulting in excreta with lower quantities of nitrogen than those of standard diets, may allow for potential environmental benefits.

The variations in the microbiota are largely determined by the growth phase, which in turn is accompanied by an evolution of the diet. The different protein levels at the same age caused slight but significant variations in some components of the intestinal microbiota. Further research is needed to ascertain whether microorganisms found in low-protein diets can be considered indicators of lower susceptibility to diarrhea in piglets.

## Figures and Tables

**Table 1:** Time sequence of events from weaning to the end of the experiment.

Growth Period	Event (Initial and Final of the Period)	Time from the Start of the Experiment (Days)	Piglet Age (Days)	Measurement of Piglet Body Weight	Fecal Sampling
	Weaning	0	25	+	–
Period I					
	Change of feed	25	50	+	T1
Period II					
	Change of housing	46	71	+	T2
Period III					
	End of post-weaning	70	95	+	T3

+ and – signs indicate that the actions have or have not been done, respectively.

**Table 2:** Growth phases based on piglet body weight and overall crude protein percentage in the diet, depending on growth phase and protein level.

Growth Phase	Protein Level (% CP)	
	High	Low
From the start of weaning to 15 kg (Period I and II)	18.5	16.5
From 15 kg to the end of the experiment (Period III)	16.5	14.5

**Table 3:** Feed ingredients, depending on the growth phase and dietary protein level.

		Protein Level			
Feed Composition, as Is ^1^		From 8 to 15 kg Body Weight		From 15 to 30 kg Body Weight	
		High	Low	High	Low
Ingredients					
Corn	%	18.8	23.7	26.6	35.0
Barley	%	20.0	20.0	15.0	15.0
Expanded wheat	%	10.0	10.0	6.00	6.00
Soybean meal (48% CP ^2^)	%	8.87	2.73	8.43	3.07
Wheat middlings, durum	%	0.00	0.00	8.13	4.60
Bakery byproducts	%	8.00	8.00	4.00	4.00
Whey powder, sweet	%	6.00	6.00	0.00	0.00
Soybean protein concentrate (65% CP)	%	5.00	5.00	2.00	2.00
Mineral vitamin premix	%	5.00	5.00	2.50	2.50
Wheat bran, soft	%	4.23	5.00	4.00	4.00
Wheat	%	4.00	4.00	8.00	8.00
Wheat middlings, soft	%	0.00	0.00	8.00	8.00
Fish meal (68% CP)	%	3.00	3.00	2.00	2.00
Dextrose	%	2.50	2.50	1.00	1.00
Monodicalcium phosphate	%	1.57	1.64	0.00	0.21
Chicory pulp, dehydrated	%	1.20	1.20	1.00	1.00
Coconut oil	%	0.96	0.83	1.16	1.04
Soybean oil	%	0.00	0.00	0.50	0.50
Acidifiers	%	0.80	0.80	0.80	0.80
DL-Methionine	%	0.05	0.12	0.18	0.25
L-Valine	%	0.05	0.17	0.00	0.08
L-lysine HCl	%	0.01	0.22	0.47	0.68
L-tryptophan	%	0.01	0.04	0.05	0.09
L-threonine	%	0.00	0.02	0.14	0.23
Calculated values					
Crude protein	%	18.5	16.6	16.5	14.6
Crude fat	%	5.00	5.00	4.50	4.50
Crude fiber	%	2.96	2.85	3.90	3.50
Ash	%	6.07	5.87	5.50	5.31
Digestible Energy	kcal kg^−1^	3472	3450	3324	3320
Metabolizable Energy	kcal kg^−1^	3289	3277	3176	3180
Net energy	kcal kg^−1^	2480	2510	2401	2447
Lysine	%	1.40	1.40	1.20	1.20
Methionine	%	0.54	0.58	0.45	0.49
Methionine + Cystine	%	0.84	0.84	0.72	0.72
Threonine	%	0.92	0.84	0.72	0.72
Tryptophan	%	0.28	0.28	0.24	0.24
Valine	%	0.98	0.98	0.86	0.84
Isoleucine	%	0.78	0.65	0.68	0.56
Calcium	%	0.65	0.65	0.59	0.62
Phosphorus	%	0.78	0.77	0.50	0.50

^1^ Amino acid and phosphorus composition is reported as total content. ^2^ Crude protein.

**Table 4:** Scores for diarrhea incidence assigned to each cage/pen on the basis of the fraction of litter suffering from diarrhea and diarrhea intensity.

Fraction of Litter ^1^ Suffering from Diarrhea	Diarrhea Intensity		
	Mild	Medium	Serious
0	0	0	0
1/3	1	4	7
2/3	2	5	8
3/3	3	6	9

^1^ 12 piglets per cage/pen.

**Table 5:** Average value of selected growth parameters during the experiment and significance of the difference between high-protein and low-protein diets (n = 6 per treatment).

	Protein Level		
Growth Parameter	High	Low	Significance of the Difference
Initial body weight (kg)	7.80	7.77	NS
From weaning to change of housing			
Period I ^1^	17.2	15.6	NS
Period II ^2^	29.8	28.0	NS
Period I + Period II	23.5	21.8	NS
Average daily gain (g)			
Period I	377	313	*p* < 0.01
Period II	599	593	NS
Period I + Period II	479	440	*p* < 0.05
Average daily feed intake (g)			
Period I	585	536	NS
Period II	1181	1138	NS
Period I + Period II	857	810	NS
Feed conversion ratio (-/-)			
Period I	1.55	1.72	*p* < 0.01
Period II	1.97	1.92	NS
Period I + Period II	1.79	1.84	*p* < 0.05
From change of housing to the end of post-weaning (period III) ^3^			
Average daily gain (g)	457	437	NS
Average daily feed intake (g)	952	952	NS
Feed conversion ratio (-/-)	2.11	2.18	NS
Final body weight (kg)	40.7	38.5	NS

^1^ Period I: From the start of the experiment until the change of diet; ^2^ Period II: From the change of diet until the change of housing; Period I + Period II: From the start of the experiment until the change of housing; ^3^ Period III: From the change of housing until the end of the experiment.

**Table 6:** Influence of dietary protein level on the diarrhea score summations. The percentages of the scores are shown in parentheses with respect to the total score, referring both to the protein level in the period and to the whole period.

	Diarrhea Score Summations ^1^			
	Protein Level			Significance of the Difference
Growth period	High	Low	Total in the Period	
Period I	91 (85.0)	16 (15.0)	107 (45.7)	
Period II	5 (83.3)	1 (16.7)	6 (2.6)	
Total (Period I + Period II)	96 (85.0)	17 (15.0)	113 (48.3)	*p* < 0.01
Period III	71 (58.7)	50 (41.3)	121 (51.7)	NS
Total score	167 (71.4)	67 (28.6)	234	

^1^ Scores were calculated as reported in Table 4. NS: not significant.

**Table 7:** Relative abundance of bacterial phyla in the piglet fecal microbiota depending on sampling time and protein level ^1,2^.

Phylum	Sampling Time and Protein Level									Protein Level			Significance Level ^3^		
	T1			T2			T3								
	High	Low	Mean	High	Low	Mean	High	Low	Mean	High	Low	Overall Mean	Sampling Time	Protein Level	Sampling Time × Protein Level
*Actinobacteria*	0.50	0.37	0.43A	0.23	0.18	0.21B	0.20	0.20	0.20B	0.31A	0.25A	0.28	**	NS	NS
*Bacteroidetes*	40.7	41.9	41.3B	49.4	50.7	50.1A	46.6	48.6	47.6A	45.6A	47.0A	46.3	**	NS	NS
*Fibrobacteres*	0.15	0.28	0.22B	0.38	0.72	0.55A	0.35	0.52	0.43A	0.29B	0.51A	0.38	**	**	NS
*Firmicutes*	54.4	50.6	52.5A	40.6	37.2	38.9B	43.0	39.7	41.4B	46.0A	42.5B	44.3	**	*	NS
*Proteobacteria*	1.55	2.40	1.97B	3.02	3.27	3.14A	2.52	3.15	2.83A	2.36B	2.94A	2.67	**	*	NS
*Spirochaetes*	0.90	2.43	1.67B	3.93	5.38	4.66A	4.25	4.83	4.54A	3.03B	4.27A	3.60	**	*	NS
*Unclassified*	1.63	1.78	1.71B	1.73	1.83	1.78AB	1.98	1.97	1.97A	1.78A	1.86A	1.82	*	NS	NS
*Verrucomicrobia*	0.00	0.08	0.04C	0.25	0.35	0.30B	0.57	0.57	0.57A	0.27A	0.33A	0.32	**	NS	NS

^1^ T1: sampling after weaning, before the change of feed, T2: sampling after the change of feed and before changing of housing, T3: sampling after the change of housing and before the end of the experiment (end of the post-weaning period). ^2^ Values of relative abundance greater than 0.1% were considered. ^3^ Means sharing common letters within the phylum are not significantly different. Capital letters refer to the main factor effects, sampling time, and protein level. Underscore letters for interaction effects are omitted. NS: not significant, *, *p* < 0.05, **, *p* < 0.01.

**Table 8:** Bacterial genera differentially abundant in fecal samples of piglets fed low- or high-protein diets. The notation (T1) indicates that the effect was present only at the sampling time T1.

Phylum	Family	Genus		Protein Level of Prevalent Abundance	
		Classes of Relative Abundance			
		>1%	0.1–1%	Low	High
*Fibrobacteres*	*Fibrobacteraceae*		*Fibrobacter*	+	
*Firmicutes*	*Clostridiaceae 1*	*Clostridium sensu stricto*			+ (T1)
*Firmicutes*	*Eubacteriaceae*		*Eubacterium*		+
*Firmicutes*	*Lachnospiraceae*	*Roseburia Blautia Clostridium XlVa*	*Lachnospiracea_incertae_sedis*, *Coprococcus, Anaerostipes*, *Ruminococcus2*, *Dorea*, *Fusicatenibacter*		+
*Firmicutes*	*Lactobacillaceae*	*Lactobacillus*		+ (T1)	
*Firmicutes*	*Peptostreptococcaceae*	*Clostridium XI*			+ (T1)
*Firmicutes*	*Erysipelotrichaceae*		*Catenibacterium*, *Turicibacter*, *Erysipelotrichaceae_incertae_sedis*		+ (T1)
*Proteobacteria*	*Succinivibrionaceae*		*Succinivibrio*	+	
*Spirochaetes*	*Spirochaetaceae*	*Treponema*	*Sphaerochaeta*	+	

**Table 9:** Composition of the fecal samples depending on sampling time (T1, T2, and T3) and protein level in the diet ^1^.

Parameter	Sampling Time									Protein Level		Significance Level ^2^		
	T1			T2			T3							
	High	Low	Mean	High	Low	Mean	High	Low	Mean	High	Low	Sampling Time	Protein Level	Sampling Time × Protein Level
Physico-chemical parameters:														
Dry matter (DM)	24.2	26.1	25.2C	26.5	27.6	27.1B	27.8	29.4	28.6A	26.2B	27.7A	**	**	NS
Organic matter (% DM)	87.6	88.8	88.2B	89.1	89.1	89.1A	89.2	89.8	89.5A	88.7B	89.2A	**	**	**
Total N (% DM)	4.53	4.08	4.30A	3.58	3.29	3.43B	3.01	2.94	2.98C	3.70A	3.44B	**	**	NS
Ammonium N (% DM)	0.61	0.56	0.59A	0.61	0.51	0.56A	0.49	0.45	0.47B	0.57A	0.51B	**	**	NS
pH	6.31	6.32	6.31B	6.59	6.55	6.57A	6.62	6.52	6.57A	6.51A	6.46A	**	NS	NS
Crude fiber (% DM)	15.4	14.8	15.1B	16.4	17.1	16.7A	17.6	17.2	17.4A	16.5A	16.4A	**	NS	NS
Hemicellulose	19.9	23.0	21.4C	25.0	27.9	26.1B	30.3	32.1	31.1A	25.0B	27.7A	**	**	NS
Cellulose	14.4	15.6	15.0B	16.5	16.6	16.6A	16.1	16.3	16.2A	15.7B	16.5A	**	*	NS
Volatile fatty acids (mmol kg^−1^ DM):														
Acetic	183	197	190A	237	214	225A	181	189	185A	200A	200A	NS	NS	NS
Propionic	62.1	60.3	61.2B	76.7	75.3	76.0A	66.1	66.1	66.1AB	68.3A	67.2A	*	NS	NS
Iso-butyric	13.5	26.6	21.1A	29.7	22.6	26.1A	18.5	20.2	19.4A	21.2A	23.1A	NS	NS	NS
Butyric	51.3	43.4	47.3A	53.7	47.2	50.5A	40.7	42.7	41.7A	48.5A	44.4A	NS	NS	NS
Isovaleric	10.7	12.1	11.4B	17.2	14.1	15.6A	15.1	12.7	13.9A	14.3A	13.0B	**	*	*
Valeric	10.1	11.3	10.7B	14.0	11.7	12.9A	10.9	10.3	10.6B	11.6A	11.1A	*	NS	NS
Isocaproic	4.38	6.13	5.26A	4.42	5.43	4.92A	3.92	4.80	4.36A	4.24A	5.46A	NS	NS	NS
Caproic	3.18	3.18	3.18A	4.10	2.48	3.29A	2.33	2.32	2.32A	3.21A	2.66A	NS	NS	NS
Total	341	361	351A	441	395	418A	344	358	351A	400A	367A	NS	NS	NS

^1^ T1: sampling after weaning, before the change of feed, T2: sampling after the change of feed and before changing of housing, T3: sampling after the change of housing and before the end of the experiment (end of the post-weaning period). ^2^ Means sharing commons letters within the phylum are not significantly different. Capital letters refer to the main factor effects, sampling time, and protein level. Underscore letters for interaction effects are omitted. NS: not significant, *, *p* < 0.05, **, *p* < 0.01.

## Data Availability

Data are contained within the article or supplementary material.

## Supplementary Materials

The following supporting information can be downloaded at: https://www.mdpi.com/article/10.3390/ani13050795/s1, Table S1: Number of interventions with parenteral therapy on piglets, from the onset of diarrhea to change of housing; Table S2: Diarrhea scores from the start of the experiment until the change of housing; Table S3: Diarrhea scores from the change of housing until the end of the experiment; Table S4. Bacterial families with relative abundance greater than 0.1% in the piglet fecal microbiota, depending on sampling time and protein level; Table S5: More abundant (>1% reads) bacterial genera in the fecal microbiota of weaning piglets.
