# Supplementary material for: Protein Content in the Diet Influences Growth and Diarrhea in Weaning Piglets
Source: Animals (Basel). 2023 Feb 22;13(5):795. doi: 10.3390/ani13050795 (PMC10000050; doi:10.3390/ani13050795)
Supplement: Supplementary file 1 [file animals-13-00795-s001.zip › animals-2228004-supplementary.pdf]

Supplemental information

Table S1. Number of interventions with parenteral therapy on piglets, from the onset of diarrhoea to change of housing (males in room 1, females in room 3). Change of housing was followed by mass oral treatment.

	High protein level						Low protein level					
	Room 1			Room 3			Room 1			Room 3		
ID_cage	1	2	3	1	2	3	6	7	8	6	7	8
Date												
19/11/2019				5			1					
20/11/2019	12	12			5							
21/11/2019	12	12	12		4	2	1					
22/11/2019	7	5	6	2	3	2	5				2	3
23/11/2019	12	7	5	1	3	3	1				2	2
24/11/2019	4	6	1	7	3	3	12	1	1			4
25/11/2019	1	5	6	3	1	2	4	3				
26/11/2019	3		1	2	3	1			1			
27/11/2019	2	2	5	5	2	2			1			
28/11/2019	4	5		1	3	4			3			
29/11/2019	2		2	1	3	3		1	1			
30/11/2019	1	3			3							
01/12/2019		6	1		2	1			1			
02/12/2019						1						
Treatment summation	162			86			37			13		

Table S2. Diarrhoea scores from the start of the experiment until the change of housing (males in room 1, females in room 3)

	High protein level						Low protein level					
	Room 1			Room 3						Room 1		
ID_cage	1	2	ID_cage	1	2	ID_cage	1	2	ID_cage	1	2	ID_cage
Date												
Faeces of normal consistency from 07/11/19 to 19/11/19												
19/11/2019				2								
20/11/2019	3	3			2							
21/11/2019	3	3	3		2	1						
22/11/2019	2	2	2	1	1	1	2					1
23/11/2019	3	2	2	1	1	1	1					1
24/11/2019	1	1	1	1	1	1	3	1	1			
25/11/2019	1	2	1	1	1	1	1	1				
26/11/2019	1	1	1	2	1	1			1			
27/11/2019	1	1	2	2	1	1			1			
28/11/2019	1	2		1	1	1			1			
29/11/2019	1		1	1	1	1			1			
30/11/2019		1			1							
01/12/2019	1	2		1	1	1						
02/12/2019*	1		1			1						

03/12/2019	1	1	1			1						1
04/12/2019						1						
Faeces of normal consistency from 05/12/19 to 23/12/19												
Score summation	56			40			14			3		

* Date of diet change

Table S3. Diarrhoea scores from the change of housing until the end of the experiment (Box # 50, 52, 54, 73, 75, 77: males, box # 56, 58, 60, 79, 81, 83: females).

	High protein level						Low protein level					
ID_BOX	50	52	54	56	58	60	73	75	77	79	81	83
Faeces of normal consistency from 24/12/19 al 27/12/19												
28/12/2019	2	6		4	6	5	4	2	1	1	1	2
29/12/2019	2	2	1	1		1	1	1	1			
30/12/2019	2	2	1	1		1	2	2	1		1	
31/12/2019	2	1	1	1	1	2	1	2	1		2	
01/01/2020		1			1	2		1	1	1		
02/01/2020	1	1	1	2	1	2		2	1	1	1	
03/01/2020	1	2	1	1		2	1	2	2		1	
04/01/2020			1			1		1	1		1	
05/01/2020												
06/01/2020												
07/01/2020			1					1				
08/01/2020												
09/01/2020			1					1	1			
10/01/2020		1	1			1		1	1			1
11/01/2020											1	
Faeces of normal consistency from 12/01/20 al 16/01/20												
Score summation	35			36			36			14		

Table S4. Bacterial families with relative abundance greater than 0.1% in the piglet fecal microbiota, depending on sampling time and protein level. T1: sampling after weaning, before the change of feed, T2: sampling after change of feed and before changing of housing, T3: sampling after the change of housing and before the end of the experiment (end of the post-weaning period). LP: low protein diet, HP: high protein diet.

Phylum	Family	Sampling time									Protein level			Significance level		
		T1			T2			T3								
		HP	LP	Mean	HP	LP	Mean	HP	LP	Mean	HP	LP	Mean	Sampling time	Protein level	Sampling time x Protein level
Actinobacteria																
	Coriobacteriaceae	0.50	0.37	0.44A	0.24	0.19	0.22B	0.18	0.18	0.18B	0.31A	0.25A	0.28	****	NS	NS
Bacteroidetes																
	Prevotellaceae	34.3	34.1	34.2AB	37.5	37.6	37.6A	31.1	32.8	32.0B	34.3A	34.9A	34.6	*	NS	NS
	Porphyromonadaceae	3.33	4.17	3.75C	5.66	6.39	6.03B	6.93	6.66	6.80A	5.31A	5.74A	5.52	****	NS	NS
	Unclassified 1	2.41	3.11	2.76B	5.17	5.58	5.37A	5.17	4.96	5.07A	4.25A	4.55A	4.40	****	NS	NS
	Unclassified 4	0.46	0.27	0.36B	0.82	0.82	0.82B	1.33	1.68	1.50A	0.87A	0.92A	0.90	***	NS	NS
	Bacteroidaceae	0.17	0.18	0.18B	0.24	0.28	0.26B	2.06	2.30	2.18A	0.82A	0.92A	0.97	****	NS	NS
Fibrobacteres																
	Fibrobacteraceae	0.14	0.30	0.22B	0.38	0.70	0.54A	0.34	0.51	0.43A	0.29B	050A	0.40	**	**	NS
Firmicutes																
	Lachnospiraceae	21.0	17.6	19.3A	11.2	9.5	10.3B	10.6	8.9	9.8B	14.3A	12.0B	13.1	****	***	NS
	Ruminococcaceae	11.9	11.4	11.6AB	10.7	10.3	10.5B	14.6	12.6	13.6A	12.4A	11.4A	11.9	**	NS	NS
	Clostridiaceae 1	5.33	2.39	3.86A	3.46	3.96	3.71A	3.97	4.35	4.16A	4.25A	3.57A	3.91	NS	NS	**
	Unclassified 2	3.54	3.40	3.47AB	3.39	2.93	3.16B	3.88	3.60	3.74A	3.60A	3.31A	3.46	NS	NS	NS
	Lactobacillaceae	1.56	6.89	4.22A	3.50	1.93	2.72B	3.17	2.91	3.04AB	2.74B	3.91A	3.33	*	**	****
	Veillonellaceae	2.85	3.03	2.94A	3.02	2.24	2.63A	0.89	1.56	1.22B	2.25A	2.28A	2.27	***	NS	NS
	Peptostreptococcaceae	3.01	1.61	2.31A	2.02	2.71	2.37A	1.52	1.33	1.42B	2.18A	1.88A	2.03	**	NS	**
	Acidaminococcaceae	2.19	2.14	2.16A	1.62	1.80	1.71B	1.92	1.91	1.91AB	1.91A	2.13A	1.93	*	NS	NS
	Erysipelotrichaceae	2.00	0.95	1.47A	0.48	0.78	0.63B	0.81	0.91	0.86B	1.10A	0.88A	0.99	***	NS	**
	Unclassified 5	0.47	0.62	0.54B	0.89	0.65	0.77B	1.23	1.15	1.19A	0.97A	0.92A	0.84	****	NS	NS
	Eubacteriaceae	0.47	0.36	0.41A	0.18	0.18	0.18B	0.17	0.18	0.17B	0.27A	0.24B	0.26	****	*	**

	Peptococcaceae 1	0.09	0.12	0.10A	0.06	0.07	0.07B	0.08	0.07	0.08AB	0.08A	0.09A	0.08	**	NS	NS
	Streptococcaceae	0.02	0.01	0.01B	0.07	0.05	0.06B	0.14	0.19	0.16A	0.08A	0.08A	0.08	****	NS	NS
Proteobacteria																
	Succinivibrionaceae	0.36	1.04	0.70A	0.99	1.21	1.10A	0.43	1.07	0.75A	0.59B	1.10A	0.85	NS	*	NS
	Campylobacteraceae	0.41	0.34	0.38B	0.61	0.61	0.61A	0.71	0.68	0.69A	0.57A	0.54A	0.56	***	NS	NS
	Unclassified 6	0.38	0.34	0.36AB	0.43	0.50	0.46A	0.22	0.28	0.25B	0.34A	0.37A	0.36	*	NS	NS
	Unclassified 7	0.09	0.15	0.12B	0.40	0.40	0.40A	0.50	0.34	0.42A	0.33A	0.28A	0.31	****	NS	*
	Desulfovibrionaceae	0.12	0.09	0.11B	0.13	0.10	0.11B	0.21	0.21	0.21A	0.15A	0.13A	0.14	****	NS	NS
	Enterobacteriaceae	0.13	0.33	0.22A	0.12	0.08	0.10A	0.07	0.15	0.11A	0.10A	0.18A	0.15	NS	NS	NS
	Sutterellaceae	0.01	0.06	0.04B	0.09	0.13	0.11A	0.09	0.11	0.10A	0.06B	0.10A	0.08	***	*	NS
	Unclassified 8	0.01	0.00	0.01B	0.05	0.04	0.05B	0.13	0.12	0.13A	0.07A	0.06A	0.06	****	NS	NS
	Oxalobacteraceae	0.02	0.04	0.03B	0.06	0.07	0.07A	0.06	0.04	0.05AB	0.05A	0.05A	0.05	*	NS	NS
Spirochaetes																
	Spirochaetaceae	0.90	2.42	1.65B	3.92	5.38	4.65A	4.24	4.84	4.54A	3.02B	4.21A	3.62	****	**	NS
Unclassified																
	Unclassified 3	1.63	1.79	1.71B	1.75	1.83	1.79AB	1.98	1.96	1.97A	1.79A	1.86A	1.82	*	NS	NS
Verrucomicrobia																
	Subdivision5_genera_incertae_sedis	0.01	0.03	0.02C	0.20	0.33	0.27B	0.50	0.51	0.51A	0.24A	0.29A	0.26	****	NS	NS

Means sharing commons letters within Family are not significantly different. Capital letters refer to the main factor effects, sampling time and protein level. Underscore letters for interaction effects are omitted. NS: not significant, *, *P<0.05*, **, *P<0.01*, ****P<0.001*, *****P<0.0001*

Table S5. More abundant (>1% reads) bacterial genera in the fecal microbiota of weaning piglets.

Phylum	Family	Genus	Mean Percentage
Actinobacteria	*Prevotellaceae*	*Prevotella*	27.2
Actinobacteria	*Ruminococcaceae*	Unclassified	7.5
Actinobacteria	*Lachnospiraceae*	Unclassified	5.0
Actinobacteria	Unclassified	Unclassified	4.4
Actinobacteria	*Porphyromonadaceae*	Unclassified	4.2
Actinobacteria	*Prevotellaceae*	Unclassified	4.1
Actinobacteria	*Clostridiaceae 1*	*Clostridium sensu stricto*	3.9
Actinobacteria	Unclassified	Unclassified	3.5
Actinobacteria	*Lactobacillaceae*	*Lactobacillus*	3.3
Actinobacteria	*Prevotellaceae*	*Alloprevotella*	3.2
Actinobacteria	*Spirochaetaceae*	*Treponema*	3.1
Actinobacteria	*Lachnospiraceae*	*Roseburia*	2.8
Actinobacteria	*Peptostreptococcaceae*	*Clostridium XI*	2.0
Bacteroidetes	*Lachnospiraceae*	*Blautia*	1.9
Bacteroidetes	*Acidaminococcaceae*	*Phascolarctobacterium*	1.8
Bacteroidetes	Unclassified	Unclassified	1.8
Bacteroidetes	*Lachnospiraceae*	*Clostridium XlVa*	1.2
Bacteroidetes	*Ruminococcaceae*	*Ruminococcus*	1.2
Bacteroidetes	*Porphyromonadaceae*	*Barnesiella*	1.1
